# Supplementary material for: Multi-View Convolutional Neural Networks in Rupture Risk Assessment of Small, Unruptured Intracranial Aneurysms
Source: J Pers Med. 2021 Mar 24;11(4):239. doi: 10.3390/jpm11040239 (PMC8064331; doi:10.3390/jpm11040239)

Supplemental Data

The process of risk stratification and inter-assessor agreements

1st Reviewer: Heung Cheol Kim, neuroradiologist with more than 20 years of experience

2nd Reviewer: Jin Pyeong Jeon, neurointerventionist with more than 6 years of experience

3rd Reviewer: Jong Kook Rhim, neurointerventionist with more than 10 years of experience

Each reviewer reviewed DSA images independently followed by comparison of high and non-high risk UIA. Jeon JP reported the inter-assessor reliability with Cohen's kappa index to Rhim JK. All disagreements and inaccuracies were resolved and monitored by Rhim JK. The Cohen's kappa values were 0.968 and 98.5%, indicating almost perfect agreement.

n = 457		Kim's decision	
		High risk	Non-high risk
Jeon's decision	High risk	173	5
	Non-high risk	2	277

Image acquisition and post-image processing

DSA procedures were conducted with the Axiom Artis zee (Siemens Healthcare, Erlangen, Germany) or the Allura Xper FD 20/20 (Philips Medical System, Best, The Netherlands) with standard injection protocols as described previously.

Detailed image acquisition protocol of 3D-DSA

1) Axiom Artis zee: A total of 18 mL of Pamiray 250 contrast agent (Iopamidol, Dong-kook Pharmaceutical, Seoul) was administered with a tube rotation time of 4.5 s. The images were reconstructed using a 256 × 256 matrix.

2) Allura Xper FD 20/20: A total of 20mL of Visipaque 320 contrast agent (Iodixanol, GE Healthcare, USA) was administered at 4 mL/s for 12 s, and 121 projections were obtained in 240° within 4 s to reconstruct 3D images. There was a 1.5 s delay in contrast filling.

Post-processing of the 3D-DSA was conducted in an independent workstation equipped with InSpace 3D software. We collected six imaging views of UIA per patient and the six images were merged to determine the patient's rupture risk. Six views were acquired including 3 planes: frontal, sagittal, and transverse. The user-selected region-of-interest (ROI) including UIA was marked in each image.

The main limitation with preprocessing is that the images were captured by different devices. To address this limitation, histogram equalization was used to obtain similar features irrespective of DSA machines. Therefore, the edges of the aneurysm are enhanced and smoothed to extract a higher number of specific features from the aneurysm.

Supplemental Figure S1. Example of rupture risk (high vs. non-high) involving small unruptured intracranial aneurysms. The white boxes indicate the regions of interest in the aneurysm.

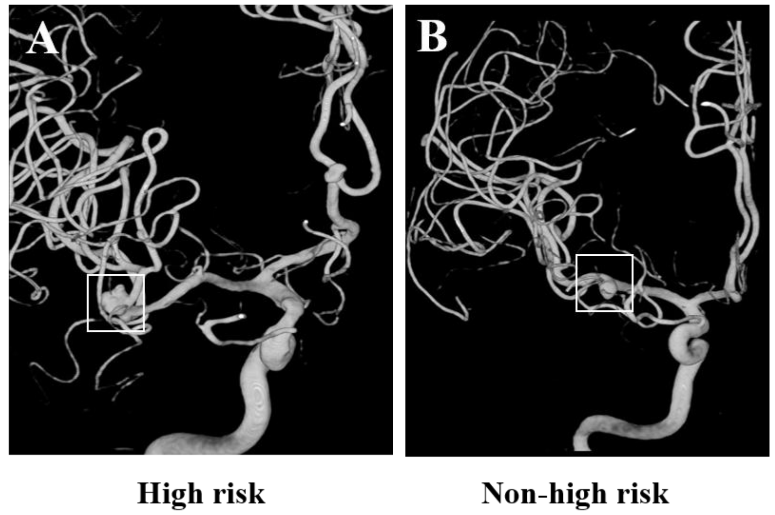

Supplemental Figure S2. Post-image processing for uniform image conversion via histogram equalization based on different three-dimensional digital subtraction angiography data

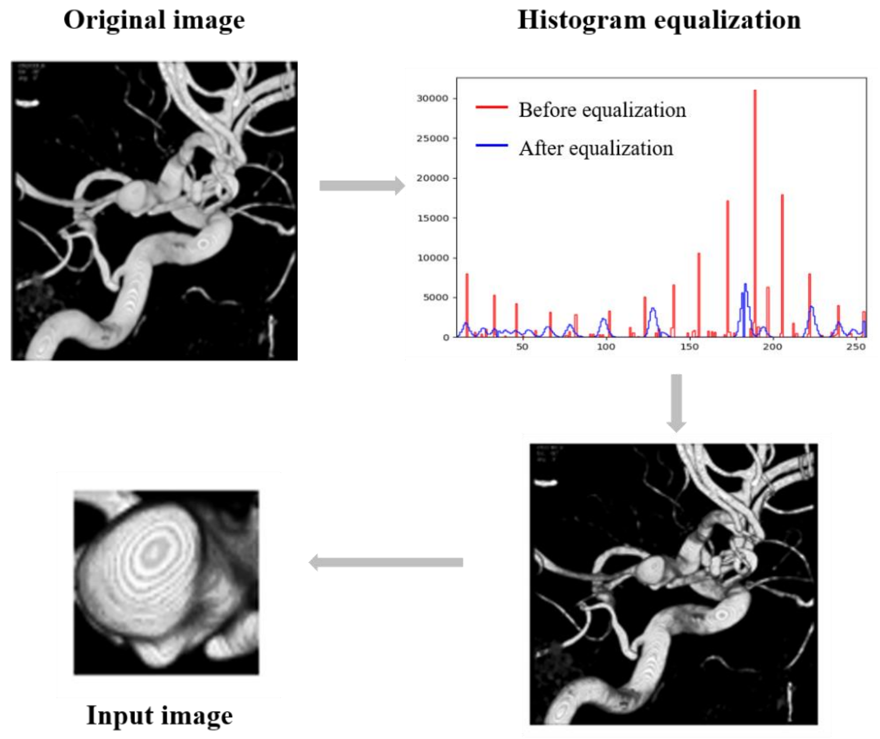

Supplemental Figure S3. Sensitivity of rupture risk differentiation (high and non-high) involving small UIAs in the training cohort using multi-view ResNet50. A sensitivity of 99.50 (95% CI: 99.10-99.89)%, a specificity of 99.96 (95% CI: 99.70-100)%, and overall accuracy of 99.63 (95% CI: 99.34-99.92)% were observed in the training cohort.

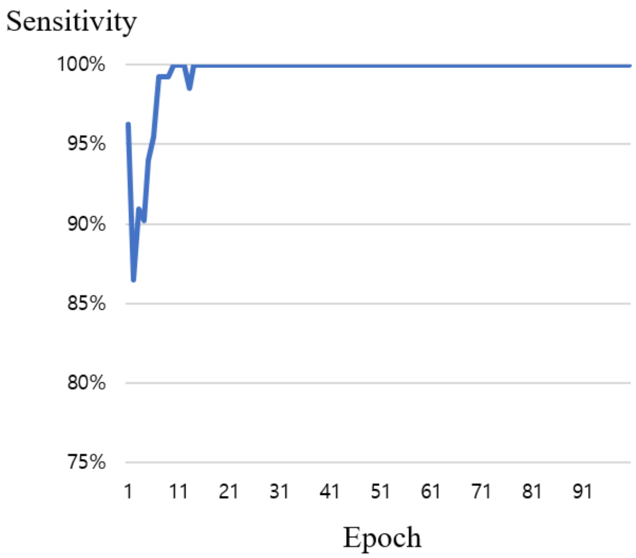

Supplement: Supplementary file 1 [file jpm-11-00239-s001.pdf]
